# Insulin-like growth factor-1 in CNS and cerebrovascular aging

**DOI:** 10.3389/fnagi.2013.00027

**Published:** 2013-07-02

**Authors:** William E. Sonntag, Ferenc Deak, Nicole Ashpole, Peter Toth, Anna Csiszar, Willard Freeman, Zoltan Ungvari

**Affiliations:** ^1^Reynolds Oklahoma Center on Aging, Department of Geriatric Medicine, University of Oklahoma Health Sciences CenterOklahoma City, OK, USA; ^2^Department of Pharmacology, Penn State College of MedicineHershey, PA, USA

**Keywords:** aging, cognitive decline, IGF-1, vasculature

## Abstract

Insulin-like growth factor-1 (IGF-1) is an important anabolic hormone that decreases with age. In the past two decades, extensive research has determined that the reduction in IGF-1 is an important component of the age-related decline in cognitive function in multiple species including humans. Deficiency in circulating IGF-1 results in impairment in processing speed and deficiencies in both spatial and working memory. Replacement of IGF-1 or factors that increase IGF-1 to old animals and humans reverses many of these cognitive deficits. Despite the overwhelming evidence for IGF-1 as an important neurotrophic agent, the specific mechanisms through which IGF-1 acts have remained elusive. Recent evidence indicates that IGF-1 is both produced by and has important actions on the cerebrovasculature as well as neurons and glia. Nevertheless, the specific regulation and actions of brain- and vascular-derived IGF-1 is poorly understood. The diverse effects of IGF-1 discovered thus far reveal a complex endocrine and paracrine system essential for integrating many of the functions necessary for brain health. Identification of the mechanisms of IGF-1 actions will undoubtedly provide critical insight into regulation of brain function in general and the causes of cognitive decline with age.

## INTRODUCTION

Cognitive decline is a common complication of aging that includes alterations in a variety of brain functions including, but not limited to, reductions in processing speed, inductive reasoning, and spatial learning and memory (Hedden and Gabrieli, 2004). Impairment of these functions is closely associated with a decrease in both health-span and independence. Over the past two decades, research studies have focused on hippocampal-dependent spatial learning and memory since they are dramatically impaired in aging subjects experiencing cognitive decline and can be important factors in disability (Hedden and Gabrieli, 2004). Age-related deficits in spatial learning and memory increase in prevalence and severity with common conditions such as type 2 diabetes, hypertension, and heart disease (Dahle et al., 2009; Okonkwo et al., 2010). Additionally, as the quality and availability of health care in developed countries continue to improve, the aged population is expected to increase (Social Science Data Analysis Network 2010). An unfortunate consequence of this is a rise in the prevalence of age-related cognitive decline. Incidence of cognitive impairment is approximately 1 in 24 at the age of 65 but increases to 1 in 3 by the age of 80 (Alzheimer’s Association). As such, greater emphasis needs to be placed on understanding, preventing, and treating cognitive impairment. The neurobiological basis of a subset of cognitive impairment, which occurs in the absence of neuronal cell death or neuropathology (Rapp and Gallagher, 1996; Rasmussen et al., 1996; Rapp et al., 2002), remains to be determined but likely involves regions of the brain associated with learning and memory including the hippocampus and prefrontal cortical regions. Within these regions impaired synaptic signaling is especially affected by aging (reviewed in Hof and Morrison, 2004) and is likely the final common pathway to cognitive impairment.

Impaired hippocampal function associated with aging occurs in many species including humans (Schaie, 1989), monkeys (Rapp and Amaral, 1989), rats (Rapp and Gallagher, 1996), and mice (Gower and Lamberty, 1993). Decreased expression of synaptic machinery, increased oxidative stress, decreased glucose metabolism, and aberrant protein folding and trafficking are characteristic structural and molecular changes that accompany this phenomenon (reviewed in VanGuilder and Freeman, 2011). Electrophysiological studies of hippocampal function demonstrate that signaling disruptions occur in animals with spatial learning and memory impairments. These electrophysiological characteristics also are associated with impaired neurotransmitter synthesis and receptor signaling, dysregulated neuronal gene and protein expression, and atypical synapse morphology (Poe et al., 2001; Shi et al., 2005; Burke and Barnes, 2006; Liu et al., 2008). Despite our understanding of the cellular changes that contribute to cognitive decline, the precise causes for the changes in brain function are unknown and represent an important challenge for neuroscientists. We, and others, have proposed that circulating factors influenced by the aging process have the potential to influence brain function either indirectly through actions on the cerebrovasculature or directly through actions on neurons and glia.

One of the factors that have profound actions on the brain is insulin-like growth factor-1 (IGF-1). Circulating IGF-1 is derived from the liver and is regulated by pulsatile secretion of pituitary growth hormone (Sonntag et al., 2005). Although IGF-1 is an important anabolic hormone throughout the body, the importance of IGF-1 in normal development of the brain is demonstrated by the striking central nervous system (CNS) phenotype of IGF-1 knockout mice. *igf-1* gene disruption results in reduced brain size, CNS hypomyelination and loss of hippocampal granule and striatal parvalbumin-containing neurons (Beck et al., 1995) suggesting that IGF-1 has a critical role in CNS development and function. Consistent with this hypothesis, transgenic mice overexpressing IGF-1 have a significantly larger brain as well as increased myelin content (Carson et al., 1993). IGF-1 has a major role in neuronal development based on studies that IGF-1 influences neuronal stem cell differentiation (Vicario-Abejon et al., 2003), axonal path finding (Scolnick et al., 2008), and dendritic outgrowth (Cheng et al., 2003; Cao et al., 2011). Studies on the role of the IGF-1 receptor are consistent with the critical effects of IGF-1 on brain development. A homozygous null mutation of the IGF-1 receptor causes neonatal lethality in mice (Liu et al., 1993; Holzenberger et al., 2003) and specific brain IGF-1 receptor knockout mice are viable but exhibit severe developmental abnormalities including dwarfism and microcephaly (Kappeler et al., 2008).

In response to decreases in growth hormone levels, IGF-1 concentrations decrease substantially with age (Sonntag et al., 2005). Importantly, studies indicate a close temporal association between the decrease in these circulating hormones and spatial and working memory performance in both rodent and human models. In humans, the importance of IGF-1 for normal body function as well as brain function has been recognized since the mid-1990s (Johansson et al., 1995; Nyberg and Burman, 1996; Burman and Deijen, 1998) and additional information regarding the relationship between IGF-1 and brain function has recently become even more apparent (Ross, 2005; Aleman and Torres-Aleman, 2009). In adults, circulating IGF-1 deficiency is associated with cognitive dysfunction (Deijen et al., 1996; Lijffijt et al., 2003; van Dam, 2005; Koltowska-Haggstrom et al., 2006) that can be reversed by increasing circulating IGF-1 levels (Sartorio et al., 1995; Deijen et al., 1998; Golgeli et al., 2004; Oertel et al., 2004; Arwert et al., 2006). Rodents have a similar decrease in circulating IGF-1 levels with age and intra-cerebroventricular (*icv*) IGF-1 replacement to older F344xBN rats, which increases concentrations of IGF-1 in the hippocampus to levels found in young animals, reverses these cognitive deficits (Trejo et al., 2007). A similar reversal of age-related memory deficits also occurs in response to peripheral administration of growth hormone (Sonntag et al., 2005) or injection of growth hormone releasing hormone (GHRH) that increases both growth hormone and IGF-1 levels (Thornton et al., 2000). The relevance of circulating IGF-1 to CNS function is perhaps best demonstrated in liver-specific IGF-1 knockout mice that exhibit a 60% reduction of IGF-1 levels at an early age (comparable to the decreases observed in aged rats and humans). These animals exhibit a reduction in learning and memory and also have a deficit in perforant path long-term potentiation (LTP; a molecular correlate of learning and memory) that results from the selective loss of excitatory inputs (Trejo et al., 2007). In addition to effects on learning and memory, IGF-1 has been shown to increase positive affective states in rodents (assessed by rough and tumble play and hedonic ultrasonic vocalizations). These studies lead to the conclusion that deficiencies in IGF-1 not only affect learning and memory but may have a role in the onset of depression (Burgdorf et al., 2010). Thus, there is now extensive evidence that in many species the age-related decrease in circulating IGF-1 is an important factor that regulates brain function as well as brain aging.

The purpose of this review is to assess the complex roles of IGF-1 in the genesis of cognitive impairment with age. Although the conclusion from numerous studies is that IGF-1 deficiency is an important contributing factor in deficits in learning and memory both in aged humans as well as rodent models of aging, a consensus for a single, specific action of IGF-1 has not emerged. Rather the data indicate that IGF-1 has both important vascular and neurotrophic actions that support multiple aspects of brain health. Here, we review data on the actions of IGF-1 on cerebrovascular structure and function, glia, and neurons and specifically on synaptic function. The emerging studies indicate that IGF-1 acts on all these cells and tissues to regulate brain health. It should be noted that this review does not address the potentially important relationship between IGF-1, age-related cognitive dysfunction and Alzheimer’s disease. Although Alzheimer’s disease is closely associated with aging and in the early stages of the disease share many behavioral similarities, the molecular mechanisms for these two conditions diverge at some point during the progression of the disease. Despite these separate mechanisms, the molecular changes that occur in the brain with age remain the primary risk factor for Alzheimer’s disease. For additional information on this topic please refer to recent reviews (Selkoe, 2012; Krstic and Knuesel, 2013; Wirth et al., 2013).

## CEREBROVASCULAR DYSFUNCTION AND COGNITIVE DECLINE IN THE ELDERLY: ROLE OF AGE-RELATED IGF-1 DEFICIENCY

Cerebrovascular alterations play a key role in various cognitive disorders in the elderly, including post-stroke dementia, multi-infarct dementia, subcortical ischemic vascular disease and dementia, mild cognitive impairment, and even Alzheimer’s disease (Gorelick et al., 2011). Cerebrovascular alterations are also likely to exacerbate cognitive decline in elderly patients with metabolic diseases, hypertension, other vascular risk factors (e.g., hyperhomocysteinemia) as well as lifestyle factors (e.g., lack of exercise). The cerebrovascular mechanisms affected by aging that promote neuronal dysfunction and cognitive decline are likely multifactorial. These include, but are not limited to, (a) impaired delivery of oxygen and nutrients to neurons (due to large vessel disease and/or structural and functional alterations of the cerebral microcirculation), (b) endothelial dysfunction and impaired neurovascular coupling, (c) impaired autoregulation, (d) disruption of the blood–brain barrier (BBB) resulting in leakage of plasma-derived pro-inflammatory factors, endothelial activation, and entry of inflammatory cells into the brain, and (e) endothelial senescence that leads to altered secretion of endothelium-derived trophic factors, impaired neurogenic niches and increased secretion of pro-inflammatory cytokines and matrix metalloproteinases from the microvascular endothelium. In addition, abnormal function of the glymphatic system, a paravascular pathway believed to be essential for clearance of solutes and waste products (including amyloid beta, Aβ) from the brain (Iliff et al., 2012), may also contribute to cognitive decline in the elderly. Despite significant advances in recent years, the mechanisms underlying age-related cerebrovascular alterations still are not completely understood (Ungvari et al., 2010b).

Investigators have recognized that circulating IGF-1 is an important vascular protective factor and that the age-related decline in IGF-1 levels contribute to vascular aging (reviewed recently in Ungvari and Csiszar, 2012). Epidemiological studies clearly indicate that growth hormone and IGF-I deficiency in humans are associated with premature atherosclerosis and increased risk for cardiovascular and cerebrovascular diseases (Rosen and Bengtsson, 1990; Bates et al., 1996; Spallarossa et al., 1996; Bulow et al., 1997; Tomlinson et al., 2001; Juul et al., 2002; Roubenoff et al., 2003; van den Beld et al., 2003; Vasan et al., 2003; Conti et al., 2004; Laughlin et al., 2004; Ungvari and Csiszar, 2012). Because of the important association between age-related cerebrovascular impairments and cognitive decline, the potential neuroprotective effects of IGF-1 are considered as partially mediated through cerebromicrovascular protection.

### CEREBROMICROVASCULAR RAREFACTION AND IMPAIRED REGIONAL BLOOD FLOW IN AGING: ROLE OF IGF-1 DEFICIENCY

Delivery of nutrients, clearance of metabolites, and exchange of gases between the blood and tissues occurs almost exclusively in the microcirculation. Thus, adequate blood perfusion via the microcirculatory network is essential for the integrity of tissues and normal organ function. Previous studies demonstrate that aging impairs regional cerebral blood flow in humans (Martin et al., 1991; Nakano et al., 2000), which certainly contributes to altered neuronal function. As the nervous system ages, there is a significant rarefaction of the microvasculature in the hippocampus and other regions of the brain involved in cognition and there are structural changes in the vessels that remain (Sonntag et al., 1997, 2000b; Khan et al., 2002; Riddle et al., 2003). It is thought that microvascular rarefaction is a major factor underlying inadequate cerebral perfusion resulting in cognitive dysfunction in the absence of, or preceding, neurodegeneration in the elderly. The decline in regional cerebral blood flow due to age-related microvascular rarefaction likely reduces metabolic support for neural signaling, especially when neuronal activity is high. In addition, aging reduces microvascular plasticity and the ability of the cerebral circulation to adapt to changes in metabolic demand (Riddle et al., 2003). Importantly, recent studies demonstrate that growth hormone supplementation, which significantly elevates circulating levels of IGF-1, substantially increases cortical vascular density in older rats (Sonntag et al., 1997) and results in significant improvements of cognitive function (Lichtenwalner et al., 2001; Poe et al., 2001; Khan et al., 2002; Ramsey et al., 2004; Sonntag et al., 2005; Hua et al., 2008). Similar increases in cerebrovascular density are observed in mice infused with IGF-1(Lopez-Lopez et al., 2004).

The mechanisms by which IGF-1 reverses/prevents microvascular rarefaction and improves tissue blood supply are likely multifaceted. On the basis of the available evidence it is possible to speculate that an increased rate of apoptosis in capillary endothelial cells contributes to age-related microvascular rarefaction. Accordingly, both in aged laboratory rodents and in non-human primates the prevalence of apoptotic endothelial cells significantly increases (Asai et al., 2000; Csiszar et al., 2004, 2007; Pearson et al., 2008), at least in part, due to impaired bioavailability of nitric oxide (NO), oxidative stress and/or chronic low-grade inflammation (Csiszar et al., 2004, 2007). One well-recognized action IGF-1 is suppression of apoptosis (Bailey-Downs et al., 2012), likely by preserving the functional integrity of the mitochondria (Li et al., 2009). Additional studies are needed to test whether augmentation of IGF-1 signaling in aging will also exert anti-apoptotic actions in endothelial cells effect resulting in the prevention or reversal of age-related cerebromicrovascular rarefaction.

Another mechanism, which potentially contributes to microvascular rarefaction is an age-related impairment of angiogenesis (Rivard et al., 1999). IGF-1 is known to confer pro-angiogenic effects, inducing proliferation of cerebromicrovascular endothelial cells via a hypoxia-inducible factor-1 alpha (HIF-1α) and vascular endothelial growth factor (VEGF)-dependent pathway (Lopez-Lopez et al., 2004). Using various animal models of age-related cerebrovascular diseases it has been reported that IGF-1, in addition to its direct neurotrophic effects, exerts angiogenic effects and protects the brain from experimental ischemic injury (Loddick et al., 1998; Guan et al., 2000, 2001; Liu et al., 2001; Schabitz et al., 2001; Mackay et al., 2003; Leinninger and Feldman, 2005). Previous studies indicate that IGF-1 has a significant role in cerebral angiogenesis both during development and in adulthood (Conti et al., 2004; Lopez-Lopez et al., 2004). Importantly, physical exercise, which is known to increase cerebromicrovascular density in control mice, fails to do so in mice with low serum IGF-1 (Lopez-Lopez et al., 2004). Previous studies demonstrated that overexpression of IGF-1 either before or after induction of cerebral ischemia enhance neurovascular remodeling, increasing cerebromicrovascular density and improving functional outcomes in rodent models of ischemic stroke (Zhu et al., 2008, 2009b). In contrast, disruption of IGF-1 signaling by an anti-IGF-1 antibody abrogates peri-lesion microvascular growth in the brain (Lopez-Lopez et al., 2004).

Age-related impairment of endothelial cell turnover due to decreased number and impaired function of endothelial progenitor cells may also negatively impact the microcirculation. Importantly, age-dependent impairment of endothelial progenitor cells was reported to be corrected by the growth hormone-mediated increase in circulating IGF-1 (Thum et al., 2007), which likely exerts beneficial effects on the regenerative capacity of the cardiovascular system in the elderly. Additionally, *in vitro* studies demonstrate that the presence of sera from young rats (which have high IGF-1 levels) in the culture medium improves the function of endothelial progenitor cells isolated from aged rats (Zhu et al., 2009a).

### VASCULAR OXIDATIVE STRESS AND ENDOTHELIAL DYSFUNCTION IN AGING

Increased oxidative stress and endothelial dysfunction are characteristics of vascular aging in general (Ungvari et al., 2010b). Earlier studies demonstrated that up-regulation of nicotinamide adenine dinucleotide phosphate (NADPH) oxidases with age promotes oxidative stress in the cerebral microvasculature (Park et al., 2007). Age-related oxidative stress impairs the bioavailability of NO, which is responsible, at least in part, for impairment of cerebromicrovascular function (Park et al., 2007) and may contribute to microvascular rarefaction. This concept is supported by studies indicating that rodents with genetically impaired NO signaling (Kubis et al., 2002) or animals treated with NO synthesis inhibitors (Frisbee, 2005) develop microvascular rarefaction in the systemic circulation. Several lines of evidence suggest that vascular oxidative stress and decreased NO bioavailability results from IGF-1 deficiency. First, animal models of IGF-1 deficiency often exhibit increased reactive oxygen species (ROS) production and decreased NO bioavailability, mimicking the vascular aging phenotype (Csiszar et al., 2008; Ungvari et al., 2010a; Bailey-Downs et al., 2012). Second, treatment of aged rats with IGF-1 up-regulates endothelial NO synthase (eNOS) and improves bioavailability of NO (Pu et al., 2008; Cittadini et al., 2009). IGF-1 treatment has similar effects in mouse models of accelerated vascular aging (Sukhanov et al., 2007). Finally, *in vitro* IGF-1 reduces ROS production and up-regulates eNOS in cultured endothelial cells (Csiszar et al., 2008).

### AGE-RELATED IMPAIRMENTS OF NEUROVASCULAR COUPLING

Neurovascular coupling is the mechanism that maintains an optimal neuronal microenvironment by adjusting local blood flow to neuronal activity. Previous studies using an event-related color-word matching Stroop task and functional near-infrared spectroscopy demonstrated that neurovascular coupling declines in the prefrontal cortex with age (Schroeter et al., 2003, 2007). Similar conclusions were reached in studies using simultaneous recording of cerebral blood flow velocity responses and visual evoked potentials using graded visual contrasts (Zaletel et al., 2005). The breakdown of the molecular communication between neurons and microvessels and the resulting uncoupling between metabolism and regional cerebral blood flow likely contribute to age-related cognitive impairment. Although the specific mechanisms underlying age-related impairment of neurovascular coupling are not completely understood, these effects are likely associated with increased oxidative stress and endothelial dysfunction (Park et al., 2007). In addition, age-related changes in astrocyte function, the extracellular matrix and innervation of the vascular wall may also contribute to age-related impairment of neurovascular coupling. IGF-1 was reported to regulate astrocyte function (Ni et al., 1997; Aberg et al., 2003), but the role of IGF-1 deficiency in the age-related impairment of neurovascular coupling remains elusive. Seminal studies by the Iadecola laboratory demonstrate that bioavailability of NO (Zhang et al., 1998; Kazama et al., 2003, 2004; Park et al., 2005, 2007; Girouard et al., 2007) determines the efficiency of neurovascular coupling. Since IGF-1 has an important role in the regulation of microvascular NO synthesis (see above), further studies are necessary to elucidate the causal link between IGF-1 deficiency and impaired functional hyperemia in aging.

### AGE-RELATED CHANGES IN AUTOREGULATION OF CEREBRAL BLOOD FLOW

Regulation of cerebral blood flow depends on a complex interaction between various regulatory mechanisms, including mechanotransduction of pressure/wall tension and shear stress, metabolic factors, chemical factors (pCO_2_, pH.pO_2_), mediators released from astrocytes and pericytes as well as neural control. Mechanisms that respond to changes in pressure and blood flow-related shear stress are responsible for autoregulation of cerebral blood flow resulting in stable, constant cerebral perfusion despite changes in systemic blood pressure. Autoregulation responds to two different, bi-directional requirements; vasodilation and a decrease in vascular resistance in the presence of decreasing blood pressure (e.g., due to orthostatic hypotension) and vasoconstriction and increased cerebrovascular resistance in response to sudden increases in blood pressure. The dilation and constriction of cerebral vessels in response to changes in systemic blood pressure is predominantly regulated by pressure- and flow-sensitive mechanisms (including 20-hydroxyeicosatetraenoic acid (20-HETE) and transient receptor potential cation channels, subfamily C, member 6 (TRPC6) channel-mediated increases in smooth muscle [Ca^2^^+^]_i_) that are intrinsic to the vascular wall. Dysfunction of cerebral autoregulation has multiple consequences. For example, inadequate dilation in response to a decrease in blood pressure can lead to ischemic damage, whereas insufficient constriction of proximal branches of the cerebrovascular tree permits increased arterial pressure to penetrate the distal portion of the microcirculation resulting in damage to the thin-walled arteriolar and capillary microvessels. Such dysfunction is thought to contribute to various pathophysiological conditions affecting the brain, including Alzheimer-disease (Niwa et al., 2002).

Several lines of evidence suggest that aging *per se* impairs autoregulation. For example, aging is associated with a higher incidence of postural syncope, a common consequence of sudden blood pressure drop in the elderly (Campbell et al., 1990). A larger postural reduction in cerebral cortical oxygenation and more pronounced decline in mean blood flow velocity in middle cerebral arteries are manifest in elderly as compared to young individuals (Mehagnoul-Schipper et al., 2000; Lucas et al., 2008). Both in elderly humans and aged rodents, failure of static autoregulation to maintain constant cerebral blood flow during hypotension has been reported (Wollner et al., 1979; Lartaud et al., 1993). Clinical studies also suggest that aging impairs autoregulatory protection mechanisms in response to high blood pressure in the human brain (Castellani et al., 2006). Studies from our own laboratory provide evidence that aged mice exhibit pathological loss of cerebral autoregulatory protection, which contributes to an exacerbation of hypertension-induced cerebromicrovascular injury (Toth, Csiszar, Sonntag, and Ungvari, 2012, unpublished observation). Downstream consequences of cerebrovascular autoregulatory dysfunction with age may include disruption of the BBB, neuroinflammation due to microglia activation, leakage of plasma-derived pro-inflammatory factors and cognitive decline (Zlokovic, 2008).

The age-related mechanism(s) that are responsible for impaired autoregulation are not well understood. The existing evidence supports the concept that in young animals activation of a 20-HETE/TRPC-dependent pathway and arterial remodeling contribute to functional adaptation of cerebral arteries to hypertension and that these adaptive responses are dysfunctional in aging. It is possible that age-related IGF-1 deficiency exerts an important role in maladaptation of cerebral arteries to changes in the hemodynamic environment (Ungvari and Csiszar, 2012). For example, recent studies demonstrate that hypertension in IGF-1 deficient mice is associated with impaired adaptive changes in cerebral arterial myogenic constriction (Toth and Ungvari, unpublished observation, 2012) mimicking the aging phenotype. IGF-1 deficiency also results in down-regulation of cytochrome P450 4A ω-hydroxylases [see Gene Expression Omnibus (GEO) datasets GDS2019 and GDS1053], which produce 20-HETE. Further, IGF-1 has been shown to regulate calcium influx through TRP-channels (Kanzaki et al., 1999) and improves microvascular autoregulation in chronic kidney disease (Lin et al., 1998). Additional studies will be required to determine whether IGF-1 treatment reverses autoregulatory defects that occur with age.

### ROLE OF BLOOD–BRAIN BARRIER DISRUPTION

The BBB is the physical and metabolic barrier between circulating blood and brain tissue; a dynamic interface necessary for proper function of the CNS. There is growing evidence that when the BBB is disrupted the unique physicochemical milieu needed for neuronal function is not maintained, which contributes to cognitive decline (Zlokovic, 2008, 2011). Neurons and glia are highly sensitive to the effects of circulating bioactive molecules that normally do not cross an intact BBB. However, after disruption of the BBB extravasation of serum components (including thrombin, plasmin, fibrinogen, and IgG) result in activation of microglia and increased production of matrix metalloproteinases, inflammatory cytokines and ROS producing damage to neurons as well as astrocytes and pericytes (Zlokovic, 2008, 2011). There is strong evidence that aging *per se* causes disruption of the BBB both in humans (Farrall and Wardlaw, 2009), laboratory rodents and non-human primates (Mackic et al., 1998) and these alterations have been proposed to contribute to chronic low-grade neuroinflammation. We have recently demonstrated that age-dependent increases in BBB permeability in mice in various regions of the brain (cortex, white matter, hippocampus) are associated with microglia activation, inflammation, and oxidative stress; an effect exaggerated by the presence of hypertension (Toth P, Csiszar A, Sonntag WE, and Ungvari Z, unpublished observation, 2012). Receptors for IGF-1 are abundantly expressed on cells that constitute the BBB and the expression of tight junction proteins necessary for proper BBB function (e.g., zonula occludens-1, ZO-1) appear to be regulated by IGF-1 in cultured cells (Ko et al., 2009). Nevertheless, the role of IGF-1 deficiency in age-related disruption of the BBB is not completely understood. Previous studies demonstrate that endothelial cell-specific knockout of the IGF-1 receptor does not lead to significant BBB disruption (Kondo et al., 2004). In contrast, our recent findings demonstrate that although liver-specific knockdown of IGF-1 (*Igf1 *^f/f^ + MUP-iCre-AAV8) does not cause BBB disruption under baseline conditions, it exacerbates BBB disruption elicited by hypertension, mimicking the aging phenotype (Toth P, Csiszar A, Sonntag WE, and Ungvari Z, unpublished observation, 2012).

## AGING OF GLIA AND THE ROLE OF IGF-1

Glial cells including astrocytes, microglia, and oligodendrocytes have a vital role in regulating neurovascular communication and neuron survival. In addition to their well-known role as a buffer of the synaptic space, astrocytes release a variety of regulatory substances that modulate both vasculature and neurons (Pfrieger and Barres, 1997; Allen and Barres, 2005; Koehler et al., 2006; Barres, 2008; Petzold and Murthy, 2011). Astrocytes contribute to the structure and integrity of the BBB and are known to regulate both cerebral blood flow as well as the flux of cerebrospinal fluid into the brain (Abbott, 2002; Anderson and Nedergaard, 2003; Abbott et al., 2006; Takano et al., 2006; Iliff et al., 2012). Because astrocytes have a key role in maintaining homeostasis within the brain, it is easy to appreciate how alterations in astrocyte function can have a dramatic impact on neuronal physiology. Recently, our lab reported an increase in reactive astrocytes in aged rats (VanGuilder et al., 2011a). This is consistent with previous studies indicating the up-regulation of glial fibrillary acidic protein (GFAP) and vimentin (both intermediate filament proteins within astrocytes) mRNA and protein expression in aged rodents and humans (Nichols et al., 1993; David et al., 1997; Porchet et al., 2003). While several aspects of astrocyte physiology remain unexplored in aged animals, cytokine production, specifically the production of tumor necrosis factor-α (TNF-α), interleukin (IL)-1β, IL-6, and monocyte chemotactic protein-1 (MCP-1), is known to be elevated in aging astrocytes (Campuzano et al., 2009; Cowley et al., 2012). Whether the loss of IGF-1 contributes to these age-related changes in astrocyte number and function is unknown; however, recent studies suggest that IGF-1 has a critical role in regulating astrocytic activity. In a model of amyotrophic lateral sclerosis (ALS), IGF-1 treatment, acting specifically through astrocytes, reduced neurotoxicity and rescued the disease-associated neurite retraction (Dodge et al., 2008). These results provide some of the first evidence that the age-induced loss of IGF-1 may dramatically influence astrocyte physiology.

Microglial cells, the resident immune cells within the brain, may also be influenced by the decreased availability of IGF-1 in aged animals. Once activated and recruited to the site of injury/damage, microglia play a critical role in removing cellular debris from the extracellular space. Interestingly, this phagocytic activity of microglia is reduced in aged animals (Sheng et al., 1998; Njie et al., 2012). We, and others, have reported an increase in the number of activated microglia within the hippocampus of aged rats (Ogura et al., 1994; Sheng et al., 1998; Mouton et al., 2002; Frank et al., 2006; Miller and Streit, 2007; VanGuilder et al., 2011a). Moreover, aging leads to increased expression of several microglial-specific major histocompatibility complex class II (MHC-II) immune response-associated genes and increased production of specific cytokines (Perry et al., 1993; Frank et al., 2006; Griffin et al., 2006; VanGuilder et al., 2011a). This increased pro-inflammatory profile has led to the assumption that microglia underlie the sensitization of the aged brain to neurodegeneration (Medzhitov, 2008; Wong, 2013). However, we find no differences between the microglia of cognitively impaired aged animals and age-matched controls (VanGuilder et al., 2011a).

Oligodendrocytes have a critical role in the regulation of neuronal excitability since they are responsible for the ensheathment of axons in myelin. Thus, alterations in oligodendrocyte function can dramatically impair neuronal signaling. We have previously reported an increase in myelination proteins in the hippocampus of cognitively impaired aged rats (VanGuilder et al., 2011b, 2012; VanGuilder Starkey et al., 2013b). This up-regulation included an increase in myelin-associated proteins on the surface of oligodendrocytes and neurons, including myelin-associated glycoprotein (MAG), myelin-oligodendrocyte glycoprotein (MOG), and neurite outgrowth inhibitor (NOGO-A), as well as an increase in the neuronal receptor complexes for these myelination proteins, Nogo-66 receptor 1 (NgR1) and co-receptors p75, TROY (a member of the Tumor necrosis factor receptor superfamily, member 19, also known as TNFRSF19) and LRR and Ig domain-containing, Nogo Receptor-interacting protein (LINGO-1) (VanGuilder et al., 2011b, 2012; VanGuilder Starkey et al., 2013b). Furthermore, we reported an age-related decrease in antagonists of the NgR1 myelination pathway (VanGuilder Starkey et al., 2013a). While aging is often associated with demyelination, genes associated with myelin turnover and the total number of oligodendrocytes have been shown to increase with age (Blalock et al., 2003; Peters and Sethares, 2004). Thus, it has been suggested that the increased proliferation of oligodendrocytes may be an attempt to compensate for demyelination (Peters and Sethares, 2004). It is possible that the up-regulation of these myelin-associated proteins observed in cognitively impaired aged animals is an aberrant effort of both the oligodendrocytes and neurons to restore oligo-neuronal communication/myelination that ultimately results in a loss of long-range cortical association pathways and synaptic efficacy. A loss of synaptic efficacy is also observed when IGF-1 is depleted. Interestingly, IGF-1 has been shown to have a protective role for oligodendrocytes, as administration of IGF-1 is associated with a reduction of oligodendrocyte apoptosis following a variety of insults (Ye and D’Ercole, 1999; Mason et al., 2000; Cao et al., 2003; Lin et al., 2005). Besides these limited studies, the influence of IGF-1 on oligodendrocytes remains largely unknown. Thus, as with the other glial cells, further work is required to delineate the impact of the age-associated decrease in IGF-1 on oligodendrocyte physiology.

## SYNAPTIC DYSFUNCTION, AGING, AND IGF-1

In both human and animal models, deficits of executive function as well as spatial learning and memory are manifest in a significant percent of the aged population. Although alterations in the function of glial cells and elements of the cardiovascular system with age certainly contribute to these effects as discussed above, there is no comprehensive understanding of how these changes result in cognitive impairment. Furthermore it is clear that there are many morphological and biochemical changes that occur in the CNS of the elderly but these changes may not be directly associated with impaired function. Interestingly, increased activity of neuronal circuits in the aged brain may have a compensatory role and could compensate (although most likely less effectively) for the loss of important cognitive tasks, as described in detail for the Scaffolding Theory of brain aging. This theory was proposed to explain the increased “frontal activation with age as a marker of the adaptive brain that engages in compensatory scaffolding [development of alternative neural circuitry] in response to the challenges posed by declining neural structures and function” (Park and Reuter-Lorenz, 2009). Studies on brains from a variety of mammalian species including humans conclude that ultimately a reduced number of synaptic connections among neurons are the most consistent correlate with aging (Brunso-Bechtold et al., 2000; Peters et al., 2008; Giorgio et al., 2010; Soghomonian et al., 2010; VanGuilder et al., 2010; Peters and Kemper, 2011) and cognitive decline (Dickson et al., 1995; Scheff et al., 2006; VanGuilder et al., 2011b). This finding was emphasized in our recent studies on the altered expression of a set of neurotransmission-regulating proteins with age (VanGuilder et al., 2010, 2011b). Even more recently, our data indicate that age-related cognitive impairment is closely associated with a specific set of synaptic proteins with roles in functional and structural plasticity (VanGuilder and Freeman, 2011). For example, we recently reported a decrease in calcium/calmodulin-dependent protein kinase II alpha (αCaMKII) expression within cognitively impaired aged rats (VanGuilder et al., 2011b). Because CaMKII plays a critical role in the induction and maintenance of LTP (Lisman, 1994; Malenka and Nicoll, 1999; Hudmon and Schulman, 2002), the loss of CaMKII activity may underlie the decreased synaptic plasticity/efficacy in cognitively impaired aged animals. Indeed, this concept is supported by studies in the αCaMKII knockout mice, which exhibit an accelerated decline of the ability to induce LTP with animal age (Kirkwood et al., 1997). Taken together these results warrant a closer investigation of the synaptic transmission and its vulnerability with age as the best candidate mechanism for cognitive decline in the elderly.

### SYNAPTIC TRANSMISSION AND ITS ROLE IN LEARNING AND MEMORY

There are three major components of the synapse: the pre-synaptic site, the synaptic cleft and the post-synaptic site. The pre-synaptic site is activated by a depolarizing action potential, which opens voltage-gated calcium channels. The result is calcium ion influx into the terminal or synaptic bouton. Elevated intracellular calcium ion concentrations trigger the exocytosis machinery that consists of calcium sensors (synaptotagmins), SNARE (soluble NSF attachment protein receptor) proteins [synaptobrevin/vesicle-associated membrane protein (VAMP), syntaxin-1, and synaptosomal-associated protein 25 (SNAP-25)] and other regulatory binding partners (rab3, rabphilin, munc13, and munc18) that are essential for the proper spatial and temporal execution of synaptic vesicle fusion at the active zone (Fujita et al., 1996; Verhage et al., 2000; Schoch et al., 2001; Washbourne et al., 2002; Deák et al., 2004, 2006, 2009; Bronk et al., 2007; Südhof and Rothman, 2009). Lipid membranes of the vesicle and the plasma membrane are forced into close proximity creating a fusion pore through which the neurotransmitter molecules are released into the cleft. Diffusion of small neurotransmitters (e.g., glutamate and gamma-aminobutyric acid, GABA) through the narrow synaptic gap allows the neurotransmitters to bind selectively to their receptors on the post-synaptic site. The two major classes of post-synaptic receptors are ionotropic (functioning as ion channels) and metabotropic (communicating with intracellular G protein signals). For the excitatory transmitter glutamate, the ionotropic receptors are divided into three groups according to specific agonists; AMPA (2-amino-3-(5-methyl-3-oxo-1,2-oxazol-4-yl)propanoic acid), NMDA (*N*-methyl-D-aspartic acid), and kainate receptors. For the inhibitory GABA transmitter, the ionotropic receptor is referred to as GABA-A-type while the metabotropic receptor is GABA-B-type. Research studies over the past several decades indicate that learning modifies synaptic strength and that these molecular changes are responsible for memory formation. This increase in synaptic strength is termed LTP (Bliss and Collingridge, 1993). LTP was initially discovered in hippocampal excitatory synaptic connections (Bliss and Lomo, 1973) but is a ubiquitous phenomenon in a wide variety of brain regions and occurs at many different synaptic connections (including inhibitory synapses; Bliss and Collingridge, 1993; Castillo et al., 2011). The opposite effect leading to a permanent decrease in synaptic strength is termed long-term depression (LTD).

Synaptic loss and/or alterations in synaptic function are considered to be the main pathological features of cognitive decline in aging. Marked alterations of both the pre- and post-synaptic structures with age were described both in human (Honer et al., 1992; Dickson et al., 1995) and animal models (Adams et al., 2008). The expression level of the pre-synaptic marker synaptophysin is decreased in the elderly, which is likely the result of synapse loss. Since synaptophysin is a critical synaptic vesicular protein, reduced levels of this protein can be alternatively interpreted as a decrease in the number of vesicles in synaptic boutons, assuming the overall number of synapses is unchanged. In addition, synaptic morphology is altered consistent with a decrease in overall synaptic function (Adams et al., 2008). Studies indicate that synapses from older animals become weaker with induction of LTD and, as a result, these animals are more susceptible to reversal of LTP at synapses in brain regions important for learning and memory (Norris et al., 1996; Kumar et al., 2007). This phenomenon appears to be partly the result of intracellular calcium signal dysregulation (Foster et al., 2001) and also is associated with levels of oxidative stress in aged neurons (Bodhinathan et al., 2010).

In addition to synaptic loss and LTD dysfunction, specific post-synaptic NMDA receptors are down-regulated in aging rats (Sonntag et al., 2000a; Adams et al., 2008; Liu et al., 2008). Learning deficits are associated with changes in NMDA receptor subunit expression in the hippocampal CA3 field (Adams et al., 2001, 2008) and these age-dependent changes can be reversed by systematic IGF-1 treatment (Sonntag et al., 2000a,b, 2005; Adams et al., 2008) suggesting that the effects of IGF-1 on learning and memory are mediated, at least in part, through modulation of synaptic function in general and, specifically, NMDA receptors.

### NEURONAL SECRETION OF IGF-1

As previously described, 70% of circulating IGF-1 is under the control of growth hormone (Sonntag et al., 2005). Interestingly, production and secretion of IGF-1 by the CNS has been also observed (Lund et al., 1986; Adamo et al., 1988; Rotwein et al., 1988; Ayer-le Lievre et al., 1991) and brain-derived IGF-1 is dependent on neuronal activity (Hughes et al., 1999). Recently an important regulatory mechanism for IGF-1 secretion has been reported (Cao et al., 2011). Examining synaptic transmission in the olfactory bulb of synaptotagmin10 (syt10) knockout mice, Cao et al. (2011) demonstrated that syt10 is essential for IGF-1 release. Their results suggest that syt10 is required to couple enhanced neuronal firing to IGF-1 release. Consequently, paracrine effects of IGF-1 augment synaptic connections and the maturation of developing neurons in the olfactory bulb. In syt10 knockout mice, mitral and granule neurons are smaller with less extensive dendritic arborization and fewer synaptic connections. Importantly, treatment with exogenous IGF-1 completely reverses the syt10 knockout phenotype. Taken together these data elegantly demonstrate that IGF-1 is produced in neurons and stored in vesicles containing syt10, which triggers exocytosis of the vesicles and IGF-1 if dendritic calcium is sufficiently elevated during rapid neuronal firing. This study supports the conclusion that IGF-1 released from neurons effectively supports synapse formation and dendritic arborization at least within the olfactory bulb.

Considering the specific IGF actions that probably depend on neuron type, the local environment and the developmental stage (see the severe CNS phenotype of the *igf1 *^-^^/^^-^ mouse described in Section “Introduction”), more experimental data are clearly required. Emerging research indicates that IGF-1 promotes maturation of neuroblasts in the sub-ventricular zone and neuronal migration to the olfactory bulb (Hurtado-Chong et al., 2009). To what extent paracrine IGF-1 is necessary for neurogenesis in other parts of the brain remains to be understood and is beyond the scope of this paper but we refer to other recent excellent reviews on this context (Llorens-Martin et al., 2008; O’Kusky and Ye, 2012).

### ACUTE SYNAPTIC EFFECTS OF IGF-1

Although the effects of IGF-1 are generally considered to result from long-term exposure, acute application of des-IGF-1 (40 ng/ml, lacking the first three amino residues from the N-terminal of the peptide, that is not necessary for IGF-1 receptor binding) increases field excitatory post-synaptic potential (EPSP) amplitudes by 40% in the CA1 field of hippocampal slices from young rats (Ramsey et al., 2005). The enhancement is selective to current through AMPA receptors, reversible and dependent on phosphoinositide 3-kinase (PI3K) pathway activation. Furthermore, IGF-1 administration acutely affects calcium currents through L- and N-type voltage-gated channels (Blair and Marshall, 1997) of cerebellar granule cells. Augmentation of current through these calcium channels is voltage-dependent and selective since P/Q and R-type channels are unaffected. The effect on N-type channels is independent of Akt signaling whereas Akt kinase activity is essential for the potentiation of L-type currents (Blair et al., 1999) through phosphorylation of the L-type alpha1 subunit at Y2122 by PI3K/Akt pathways (Bence-Hanulec et al., 2000). Our preliminary data on hippocampal neurons offers further insight into the acute effect of IGF-1 on synaptic transmission. We have found that recycling of synaptic vesicles is significantly increased after application of des-IGF-1 (50 ng/ml; Deak, Ungvari and Sonntag, unpublished results). This is the first indication of direct enhancement of pre-synaptic neurotransmitter exocytosis by IGF-1. Further mechanistic insight at the molecular level will clarify the role of IGF-1 in synaptic transmission.

## POTENTIALLY ALTERNATIVE ACTIONS OF IGF-1 ON BRAIN AGING: IGF-1-DEFICIENT DWARF ANIMALS

Despite the numerous primary studies and reviews detailing the importance of adequate levels of circulating IGF-1 for healthy aging, the role of this potent anabolic hormone in the genesis of the aging phenotype remains controversial. Initially, the age-related decrease in IGF-1 was considered to contribute to many aspects of aging including, but not limited to, accumulation of fat mass, cardiovascular dysfunction, as well as the decline in immune function, cellular protein synthesis, and muscle mass. Later studies (as reviewed above) indicated that reduction in levels of IGF-1 has an important role in the decline in cognitive function and increased risk for neurodegenerative diseases and stroke. Despite the clear evidence that IGF-1 deficiency is a contributing factor in specific aging phenotypes, subsequent studies revealed that some, but not all, rodent models with impaired IGF-1 signaling exhibit an increased life span. Based on studies initially conducted in invertebrate organisms, such as *Caenorhabditis elegans* and *Drosophila melanogaster* (Kenyon et al., 1993; Kimura et al., 1997; Kenyon, 2001), the corresponding data in mutant and transgenic mouse models supported the conclusion that IGF-1 signaling is part of a “conserved mechanism of aging” with decreased levels of IGF-1 delaying, rather than accelerating, the aging process (Brown-Borg et al., 1996; Bartke and Brown-Borg, 2004). Thus, two disparate concepts evolved and remain present in the literature (a) that the presence of normal levels of IGF-1 accelerate aging and these absence of IGF-1 or disruption of the IGF-1 signaling pathways exert “anti-aging” effects and (b) that the age-related decline in IGF-1 contribute to the deterioration of physiological function and replacement of these hormones delay or reverse the aging phenotype. A thorough discussion of these competing viewpoints of IGF-1 action is found in recent reviews (Deak and Sonntag, 2012; Sonntag et al., 2012; Ungvari and Csiszar, 2012). Unfortunately, the primary barriers to reconciling the disparate views of IGF-1 are that they challenge our understanding of the relationship between pathology and aging, the nature of conserved mechanisms of aging, and the importance of “life span” changes in the levels of these hormones that occur throughout the life span. These conceptual differences have been exacerbated by numerous studies that draw conclusions related to aging and life span based on a low number of experimental animals, suboptimal animal husbandry, and/or the absence of end-of-life pathology to corroborate the conclusions. The result is a plethora of studies that provide varying levels of support for the hypothesis that IGF-1 deficiency increases life span.

Importantly, the role and relevance of circulating IGF-1 for brain aging has also produced conflicting results. In mice exhibiting a deficiency in circulating IGF-1, brain IGF-1 levels are actually normal and these animals appear to have normal cognitive function (Kinney et al., 2001; Sun et al., 2005). The specific source of brain IGF-1 has not been thoroughly investigated but likely is derived from the vasculature, neurons, or glia. Nevertheless, these results providing compelling support for the conclusion that paracrine-derived IGF-1 is induced in these models and support normal function of the brain into old age. Furthermore, the generally accepted interpretation that IGF-1 deficiency is a conserved pathway for aging and that animals with a deficiency in circulating IGF-1 exhibit a reduction in IGF-1 levels at the tissue level need to be reconsidered. These are areas for future research studies.

## CONCLUSION

IGF-1 has profound actions on the cerebrovasculature, glia and neurons yet we are just beginning to understand the complex effects of this hormone and the role of IGF-1 in managing the important interactions that occur between these cell types. To date, the importance of adequate levels of IGF-1 for maintenance of brain health has been established for rodents, non-human primates, and humans, supporting the fundamental role of IGF-1 in aging and, more specifically, cognitive decline. Additionally, we are just beginning to understand the role of IGF-1 deficiency in hypertension-induced pathology in the vascular system. Despite the wealth of information that is currently available, many questions remain. For example, the regulation of paracrine IGF-1 secretion (as noted in the previous paragraph) is only beginning to be understood and the relationship between circulating IGF-1 and the local production of IGF-1 remain unknown. The sources of IGF-1 that regulate brain function remain vague and the conditions that regulate IGF-1 secretion from these sources have not been established. The diverse actions of IGF-1 on multiple brain tissues and systems demonstrate that brain aging and the resulting decline in cognitive function cannot be viewed through the lens of a single cell type or tissue. Rather, only by investigating the interactions between endocrine, vascular and brain will we understand the etiology of cognitive decline with age.

## Conflict of Interest Statement

The authors declare that the research was conducted in the absence of any commercial or financial relationships that could be construed as a potential conflict of interest.
